# Peripheral oxytocin levels are linked to hypothalamic gray matter volume in autistic adults: a cross-sectional secondary data analysis

**DOI:** 10.1038/s41598-023-50770-5

**Published:** 2024-01-16

**Authors:** Raoul Haaf, Marie-Luise Brandi, Laura Albantakis, Juha M. Lahnakoski, Lara Henco, Leonhard Schilbach

**Affiliations:** 1 Independent Max Planck Research Group for Social Neuroscience, Max Planck Institute of Psychiatry, Munich, Germany; 2 Graduate School, Technical University of Munich, Munich, Germany; 3 Department of Psychiatry and Psychotherapy, Charité - Universitätsmedizin Berlin, corporate member of Freie Universität Berlin and Humboldt- Universität zu Berlin, Berlin, Germany; 4 Outpatient and Day Clinic for Disorders of Social Interaction, Max Planck Institute of Psychiatry, Munich, Germany; 5 International Max Planck Research School for Translational Psychiatry, Munich, Germany; 6 Department of Psychiatry and Psychotherapy, Ludwig-Maximilians-Universität München, Munich, Germany; 7 Institute of Neurosciences and Medicine, Brain and Behaviour (INM-7), Research Center Jülich, Jülich, Germany; 8 Institute of Systems Neuroscience, Medical Faculty, Heinrich Heine University Düsseldorf, Düsseldorf, Germany; 9 Graduate School of Systemic Neurosciences, Munich, Germany; 10 Ludwig-Maximilians-Universität München, Munich, Germany

**Keywords:** Neuroscience, Medical research

## Abstract

Oxytocin (OXT) is known to modulate social behavior and cognition and has been discussed as pathophysiological and therapeutic factor for autism spectrum disorder (ASD). An accumulating body of evidence indicates the hypothalamus to be of particular importance with regard to the underlying neurobiology. Here we used a region of interest voxel-based morphometry (VBM) approach to investigate hypothalamic gray matter volume (GMV) in autistic (n = 29, age 36.03 ± 11.0) and non-autistic adults (n = 27, age 30.96 ± 11.2). Peripheral plasma OXT levels and the autism spectrum quotient (AQ) were used for correlation analyses. Results showed no differences in hypothalamic GMV in autistic compared to non-autistic adults but suggested a differential association between hypothalamic GMV and OXT levels, such that a positive association was found for the ASD group. In addition, hypothalamic GMV showed a positive association with autistic traits in the ASD group. Bearing in mind the limitations such as a relatively small sample size, a wide age range and a high rate of psychopharmacological treatment in the ASD sample, these results provide new preliminary evidence for a potentially important role of the HTH in ASD and its relationship to the OXT system, but also point towards the importance of interindividual differences.

## Introduction

Autism Spectrum Disorder (ASD) is a pervasive neurodevelopmental condition characterized by qualitative impairments in social interaction, communication, and restricted and repetitive behavior patterns with a median global prevalence of 1% across all ages^1–3^. Symptoms can vary greatly in the individual and are often associated with other comorbidities^4,5^ (e.g. depression, alexithymia, anxiety, obsessive-compulsive disorder) and intellectual impairment^3^. Particularly in individuals with high-functioning autism (HFA) the high comorbidity rate further complicates diagnostics and prognostics, which still rely mainly on patient observation and review of medical history^4^. An important goal in recent ASD research has therefore been to develop observer-independent markers to improve the diagnostic process and tailor treatment recommendations to specific subgroups to optimize support^6^. Oxytocin (OXT) is known to modulate social behavior and cognition and has, thus, been expected to be a potential therapeutic agent for autistic individuals^7^. In light of this, research has also explored the potential role of the OXT system in ASD's pathophysiology or as a diagnostic marker^8–10^. However, the underlying neurobiology remains unclear, and reviews and meta-analyses on OXT treatment efficacy in children and adults yield mixed results^11–15^. The inability to effectively stratify subgroups within the autistic spectrum is considered a key factor leading to unsuccessful outcomes of clinical trials for ASD pharmacotherapy^16^. Recent approaches to comprehend the neurobiology of ASD and characterize individuals with ASD more effectively have employed structural neuroimaging studies, revealing diverse structural brain differences among autistic individuals compared to controls. While it has become increasingly evident that there is no single defining neuroanatomical feature of ASD, meta-analyses and reviews have suggested that there may be neuroanatomical alterations that are, at least in parts, characteristic for ASD^5,17,18^. Consequently, there is hope that regional patterns of neuroanatomical differences could serve as diagnostic, prognostic, and treatment-determining markers^18^. With regard to a potential brain structural marker of the OXT system, the hypothalamus (HTH) is of particular interest. In the central nervous system, OXT and the related hormone vasopressin (VP) are synthesized in the HTH in the magnocellular and parvocellular neurons of the supraoptic nucleus (SON) and paraventricular nucleus (PVN). Apart from axonal transport via the hypothalamo-hypophysial tract to the posterior pituitary lobe, the nuclei also project to a variety of brain regions such as the hippocampus, the amygdala and the nucleus accumbens^19^. In addition, distribution of OXT also occurs in the form of neurosecretion directly from dendrites and somata and likely also by secretion into the cerebrospinal fluid of the adjacent 3rd ventricle^20–22^. The OXT receptor has been reported to be expressed throughout the brain, particularly in the HTH and in structures of the limbic system, as well as in various cortical areas associated with social and emotional processes^23–26^. Thus, the anatomy of the OXT system is well compatible with its ascribed function to orchestrate socioemotional processes. Lesions of the HTH have been associated with a range of behavioral and emotional symptoms such as aggressiveness, depression, and social withdrawal^27^. For example, craniopharyngeoma patients, who frequently suffer from a lesion of the HTH caused either directly by the tumor or indirectly by therapeutic resection of the tumor, were found to have a high prevalence of socio-behavioral impairments^28,29^. In accordance with the hypothesis that disruptions in OXT regulation may contribute to these symptoms in craniopharyngeoma patients, there have been reports of reduced OXT levels correlating with the extent of hypothalamic damage^30^ along with significantly heightened levels of autistic traits and increased difficulties in rapid emotion recognition compared to controls^31^. This raises the question whether the socioemotional characteristics found in ASD could be similarly related to OXT and structural alterations in the HTH. Indeed, three studies which have reported structural findings of the HTH in ASD, have concordantly reported a reduced gray matter volume (GMV) or concentration in autistic children, adolescents and young adults compared to neurotypical control subjects in the corresponding age range^32–34^. In line with structural HTH alterations in autistic individuals, studies in healthy carriers of OXTR variants associated with an increased likelihood of ASD have found a significant decrease in GMV in the HTH in healthy carriers of the rs53576 and rs2254298A alleles^35,36^, for review see^37^.

Based on these previous findings, the current study sought to investigate the morphological characteristics of the HTH and its relationship to OXT, as well as its relationship to autistic traits in a sample of autistic adults without intellectual impairment and matched neurotypical controls. As a secondary analysis of cross-sectional data obtained from a previous study that investigated OXT and cortisol levels in both autistic and non-autistic adults^38^, our objectives in this study were threefold: Firstly, we aimed to assess whether there were differences in hypothalamic volume between autistic and neurotypical adult individuals. Secondly, we sought to determine whether potential distinctions in the OXT system in autism were mirrored in the structure of the HTH and whether any variations in hypothalamic volume could be attributed to OXT. Thirdly, our goal was to explore potential correlations between hypothalamic volume and autistic traits. To accomplish these objectives, we conducted a region-of-interest (ROI) analysis using voxel-based morphometry (VBM) within the hypothalamic region. We employed three distinct models: the first to compare GMV between autistic and non-autistic adults, the second to investigate potential differences in the relationships between hypothalamic GMV and peripheral OXT levels, and the third to examine the associations between hypothalamic GMV and Autism Spectrum Quotient (AQ) scores as a measure of autistic traits.

## Material and methods

### Participants

Data were assessed as part of a previously published study examining OXT and cortisol levels in autistic and non-autistic adults^38^. Recruitment sources included the “Outpatient and Day Clinic for Disorders of Social Interaction” at the Max Planck Institute of Psychiatry for autistic individuals and an online study application system on the Institute’s website, as well as public advertisements for participants in the CG (Comparison Group). The study protocol followed the guidelines of the Declaration of Helsinki and was approved by the ethics committee of the Ludwig-Maximilians-University of Munich. All participants gave written informed consent before participating in the study and received fixed monetary compensation at the end of the experiment. General exclusion criteria were severe somatic illness, a current or previous schizophrenia diagnosis, breastfeeding, pregnancy, hormonal contraception, and a contraindication to MRI. Of the sixty-four participants included in the primary study^38^, fifty-nine underwent structural MRI. After exclusion of three scans following a quality check protocol (see Supplementary information), the final dataset included brain scans from fifty-six adults aged 18–60 years: twenty-nine autistic adults in the ASD group (17 men; mean age = 36.03 ± 11.0 years) and twenty-seven non-autistic adults in the CG (9 men; mean age = 30.96 ± 11.2 years). Demographic data are reported in the results section.

### Diagnostic procedure for ASD

Autistic individuals met DSM-5 criteria for ASD and were diagnosed in accordance with current guidelines^39^. This included a diagnostic interview by a psychologist or psychiatrist with experience in diagnosing ASD that focused on DSM-5 criteria for ASD across the lifespan and, when possible and with the patient's consent, included anamnestic information from third parties (e.g., parents, siblings). In addition, autistic participants were evaluated using the Autism Diagnostic Observation Schedule-2 (ADOS-2)—Module 4^40^ (Data reported in^38^). Autistic participants had no intellectual impairment (IQ scores > 70) and were thus regarded as individuals with HFA.

### Experimental procedures and analyses

#### Questionnaires

For the quantification of autistic traits, the Autism Spectrum Quotient AQ^41^ was used. The AQ is a well-established, self-report questionnaire that provides a scaled measure of the characteristics associated with ASD on a scale of 0 to 50. The autistic traits themselves are regarded as a dimensional construct, which reflects both the autistic and the non-autistic population^42,43^. AQ scores were available for n = 55 participants (ASD: n = 28; CG: n = 27). Assessment further included completion of the Edinburgh handedness inventory^44^, a test of verbal IQ (the Wortschatztest, WST^45^) as well as a basic questionnaire to assess medication, body-mass-index (BMI) and a dichotomous assessment of lifestyle factors such as regular exercise, regular alcohol or nicotine consumption (each defined as ‘yes’ for a reported frequency of > 1/week).

#### Oxytocin quantification

While the primary study^38^ assessed plasma OXT levels before and after a physical exercise, we here focused exclusively on OXT levels in plasma at rest (i.e., baseline). For a more detailed description of sample acquisition and OXT extraction please refer to the relevant publication^38^ and in the supplementary information. In brief, the participants were asked to abstain from food (> 12h), water (> 1h) and sports the day before the study. If autistic participants took any regular psychiatric medication, they were asked not to take the medication in the morning before the OXT measurements, but after the experiments. After arriving at the outpatient unit of the MPIP at 8:30 am, blood samples were obtained at rest. OXT concentrations were quantified in an external laboratory (RIAgnosis, Sinzing, Germany) using radioimmunoassay (RIA) as previously described^46^. Data on OXT levels were available for n = 53 participants (ASD: n = 26; CG: n = 27).

#### Magnetic resonance image (MRI) acquisition

Structural brain scans were obtained using a 3 Tesla MRI scanner by GE, model ‘Discovery MR750’. 3D anatomical T1-weighted images were acquired using a FSPGR BRAVO sequence with the following parameters: Flip angle 12°, Prep Time 450, echo time (TE) 2.3 ms, frequency 256, voxel resolution 1 × 1 × 1 mm.

#### Image pre-processing and voxel-based morphometry (VBM)

All images were processed and analysed using the CAT12 toolbox (C. Gaser, Structural Brain Mapping Group, Jena University Hospital, Jena, Germany; http://dbm.neuro.uni-jena.de/cat/) implemented in SPM12 (Wellcome Trust Centre for Neuroimaging). Pre-processing was carried out using the standard pipeline and pre-set parameters as suggested in the CAT12 manual (http://www.neuro.uni-jena.de/cat12/CAT12-Manual.pdf) and involved bias field inhomogeneity correction and denoising, using the Spatially Adaptive Non-Local Means (SANLM) Filter^47^, segmentation into gray matter (GM), white matter (WM) and cerebrospinal fluid (CSF) in accordance with the unified registration approach^48^ and spatial normalization and affine registration to MNI space using a template for high-dimensional DARTEL registration derived from 555 healthy subjects of the IXI- database (http://brain-development.org/) with a final voxel size of 1.5 × 1.5 × 1.5 mm. In version 12.7 of CAT12 used here, this process is extended by refined voxel-based processing using adaptive maximum a posteriori (AMAP) estimation, a Markov Random Field approach (MRF)^49^ and accounting for partial volume effects^50^. Finally, segmentations were modulated by multiplication with the Jacobian determinant derived from spatial registrations. This step preserves the original volumes within a voxel, which are altered during registration and is recommended by default^51^. For a more detailed description of the individual steps in Cat12 we refer to the publishers website. Prior to smoothing, images were checked for correct pre-processing in accordance with the quality check protocol suggested in the Cat12 manual (see supplementary information). Following suggestions of applying comparably small kernels for analyses in the HTH due to its small size and size of expected effects^33,52^ images were smoothed with a Gaussian kernel of 4 mm (FWHM). An absolute gray matter threshold masking of 0.1 was applied to account for a possible misclassification of tissues. For statistical analyses the smoothed and modulated GM images were used. The HTH mask for the region of interest (ROI)- based VBM analyses was derived from the subcortical brain nuclei atlas (https://neurovault.org/collections/3145/)^53^. The mask was resliced to fit the template space in SPM12 and encompassed 1085 voxels.

### Statistics

Data processing and statistical analyses of demographic data, questionnaire data, OXT levels and extracted estimates from MRI images (described in the section below) were performed in SPSS (IBM Corp. Released 2020. IBM SPSS Statistics for Windows, Version 27.0. Armonk, NY: IBM Corp). Test for normal distribution of demographic data using a Shapiro–Wilk test revealed a non-normal distribution for age and the Edinburgh handedness score. Group comparison for these variables was, therefore, performed with a Man-Whitney U test, whereas Chi^2^ tests were used for categorical variables and t-tests for continuous variables. To confirm that the present subsample of participants showed OXT characteristics similar to the sample included in the primary study^38^, univariate analyses were performed to test for effects of diagnostic group on OXT levels, including age and sex as covariates. To explore possible correlations of OXT with ASD symptomatology, a pearson correlation with the plasma OXT levels and AQ scores was computed.

### Statistical image analysis

VBM analyses were performed using the general linear model (GLM) implemented in the CAT12/SPM12 statistical module. Potential variance due to age, sex and total intracranial volume (TIV) was corrected for in all analyses. Clusters were regarded as significant when falling below an initial uncorrected voxel threshold of 0.001 and an FWE-corrected cluster threshold of 0.05. Since our goal was to focus on the HTH, we computed three VBM analyses inside the HTH-ROI: We first tested for group differences, i.e. increases and decreases of hypothalamic GMV using a t-statistic. Second, to test for group differences in associations of hypothalamic GMV and OXT, i.e. interaction effects, OXT concentrations were included in a full-factorial model and tested for significant positive and negative contrasts, i.e. (GMV[ASD] × OXT > GMV[CG] × OXT) and the other way round. Third, to test if hypothalamic GMV was associated with autistic traits in autistic and non-autistic individuals, a regression analysis on all subjects was performed with AQ scores as covariate of interest and tested for significant positive and negative associations. Since we did not find a significant results in this analysis, we subsequently tested associations of GMV and AQ scores in both groups separately. To get a better impression of the nature of associations in significant clusters, we used Marsbar^54^ to extract the mean contrast estimate in significant clusters (FWE *p* < 0.05 at cluster level) to plot the distribution of contrast estimates across participants. Finally, to examine the reliability of significant results, we performed two additional analyses: First, to check if significant results observed at the voxel level were consistent at the level of overall mean hypothalamic GMV, we extracted the unadjusted eigenvariate inside the HTH-ROI for each participant to re-ran significant statistical models in SPSS. Second, to assess the regional specificity of VBM findings inside the ROI, we re-ran significant models in a whole-brain analysis. Following recommendations in the Cat 12 manual, correction for age, sex and TIV was achieved by including these variables as nuisance parameters in the respective model designs and subsequent check for design orthogonality. Since in the second model (including OXT levels) the check for design orthogonality pointed towards a co-linearity between TIV and OXT (cos (θ) = r = − 0.35) we again adhered to the CAT12 manual's guidance and implemented TIV correction in this model using global scaling with TIV.

### Ethics approval and consent to participate

All study participants provided written informed consent. Ethical approval was granted by the Ethics Committee of the Ludwig-Maximilians-University (LMU) Munich (Project number: 712-15). All procedures were performed in accordance with the Declaration of Helsinki. Participants could withdraw from the study at any time and were financially compensated for their time.

## Results

### Demographic data

Demographic and clinical data are summarized in Table 1. There were no significant differences (all *p* > *0.05*) between groups with regard to handedness, verbal IQ, BMI, or lifestyle factors. The ASD group had more males and slightly older participants compared to the comparison group, however these differences did not reach statistical significance (*p* = *0.06* for both). Thirteen subjects in the ASD group took psychiatric medication regularly.

**Table 1:** Demographic and clinical data.

	CG (n = 27)	ASD (n = 29)	Group comparison	
	Mean (± SD)/N (%)	Mean (± SD)/N (%)	t/χ^2^/U-test	*p*
Age	30.96 (11.2)	36.03 (11.0)	275.5	.06
Sex (male:female)	9:18	17:12	3.60	.06
BMI^a^	22.61 (4.1)	24.2 (4.3)	− 1.39	.17
Handedness score^a^	89.62	66.10	281.50	.13
Smoker	2 (7.40%)	5 (17.24%)	1.24	.27
Alcohol	10 (37.03%)	12 (41.37%)	0.11	.74
Exercise	20 (74.07%)	17 (58.62%)	1.49	.22
Psychiatric medication	0 (0%)	13 (44.82%)	15.76	< .001
Autism spectrum quotient (AQ)^b^	13.89 (5.47)	35.07 (10.27)	9.5	< .001
Verbal IQ (WST)	33.59 (5.23)	33.64 (3.20)	− 0.04	0.11

*ASD* Autism spectrum disorder (group); *CG* Comparison group; *SD* Standard deviation; *WST* Wortschatztest.

^a^Data available for 54 participants.

^b^Data available for 55 participants.

### Autistic traits

Comparison of AQ scores between the ASD group (M = 35.07 ± 10.27) and the CG (M = 13.89 ± 5.47) showed significantly higher scores in the ASD group, t(53) = 9.5, *p* < 0.001 (Table 1). Scores were in line with the corresponding reference norms for autistic and non-autistic individuals^43^.

### Group comparison of peripheral OXT concentrations

Similarly to the primary study^38^, the present sub-sample showed no significant main effects of diagnostic group (F(1,49) = 0.104, *p* = 0.749), sex (F(1,49) = 0.769, *p* = 0.385) or age (F(1,49) = 0.058, *p* = 0.810) on plasma OXT levels.

### Correlation of peripheral OXT concentrations and autistic symptomatology

There was no significant correlation between OXT levels and AQ scores in the overall sample (r =  − 0.05, *p* = 0.70). The same was true when correlations were determined for the two groups separately (ASD: r =  − 0.28, *p* = 0.18; CG: r = 0.08, *p* = 0.68).

### Group comparison of hypothalamic GMV

Voxel-wise group comparison of hypothalamic GMV showed no significant differences.

### Correlation of hypothalamic GMV and peripheral OXT levels

**Figure 1:**
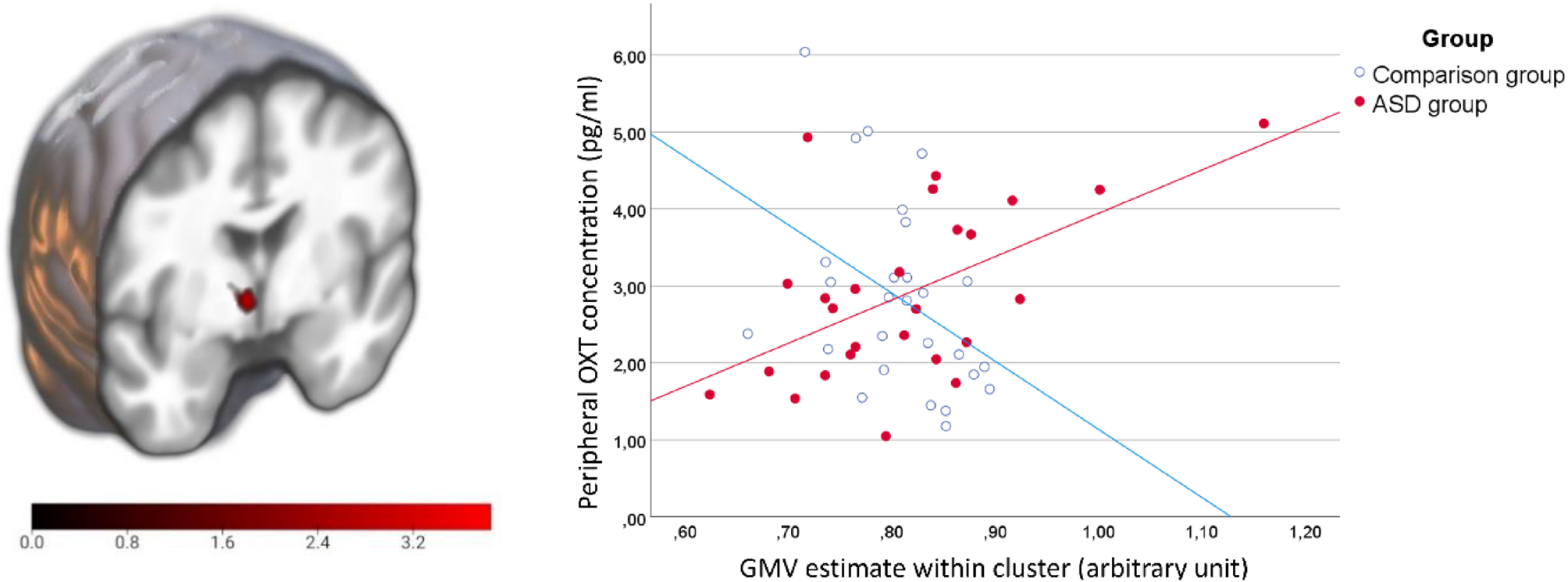
Group-comparison of associations between GMV and OXT within the HTH revealed a significant cluster (at peak-MNI coordinates [5, − 9, − 2], FWE corr. *p* = 0.017, T = 3.85, Z = 3.57, k = 46) positive for autistic adults and negative for non-autistic adults. **Left:** T-score overlay on the mean structural normalized image of all participants. **Right**: Scatterplot of the extracted GMV cluster illustrates the association with peripheral OXT in this region. Regression lines show a negative association of GMV and OXT in the CG opposed to a positive association in the ASD group. *ASD* Autism spectrum disorder; *GMV* Gray matter volume; *OXT* Oxytocin; *CG* Comparison group.

Voxel-wise group comparison of associations between hypothalamic GMV and OXT revealed significant differences in associations for the contrast (GMV[ASD] × OXT > GMV[CG] × OXT) in a cluster inside the HTH-ROI at peak-MNI coordinates [5, − 9, − 2] (FWE corr. *p* = 0.017, T = 3.85, Z = 3.57, k = 46). Plotting of the extracted GMV estimates indicated significance due to a negative association of GMV and OXT in the CG opposed to a positive association in the ASD group (Fig. 1). Similarly repetition of the statistical model using the mean hypothalamic GMV was significant for the interaction term of group and OXT (F(1, 46) = 5.675, *p* = 0.021, η^2^ = 0.110). Exploratory whole brain analysis to asses regional specificity of this finding using the same model revealed a larger cluster including the HTH and extending to the thalamus with peak-MNI coordinates at [5, − 21,9] (FWE corr. *p* = 0.005, T = 4.69, Z = 4.23, k = 1373) (Supplementary Fig. 1). No other region reached significance here.

### Correlation of hypothalamic GMV and autistic traits

**Figure 2:**
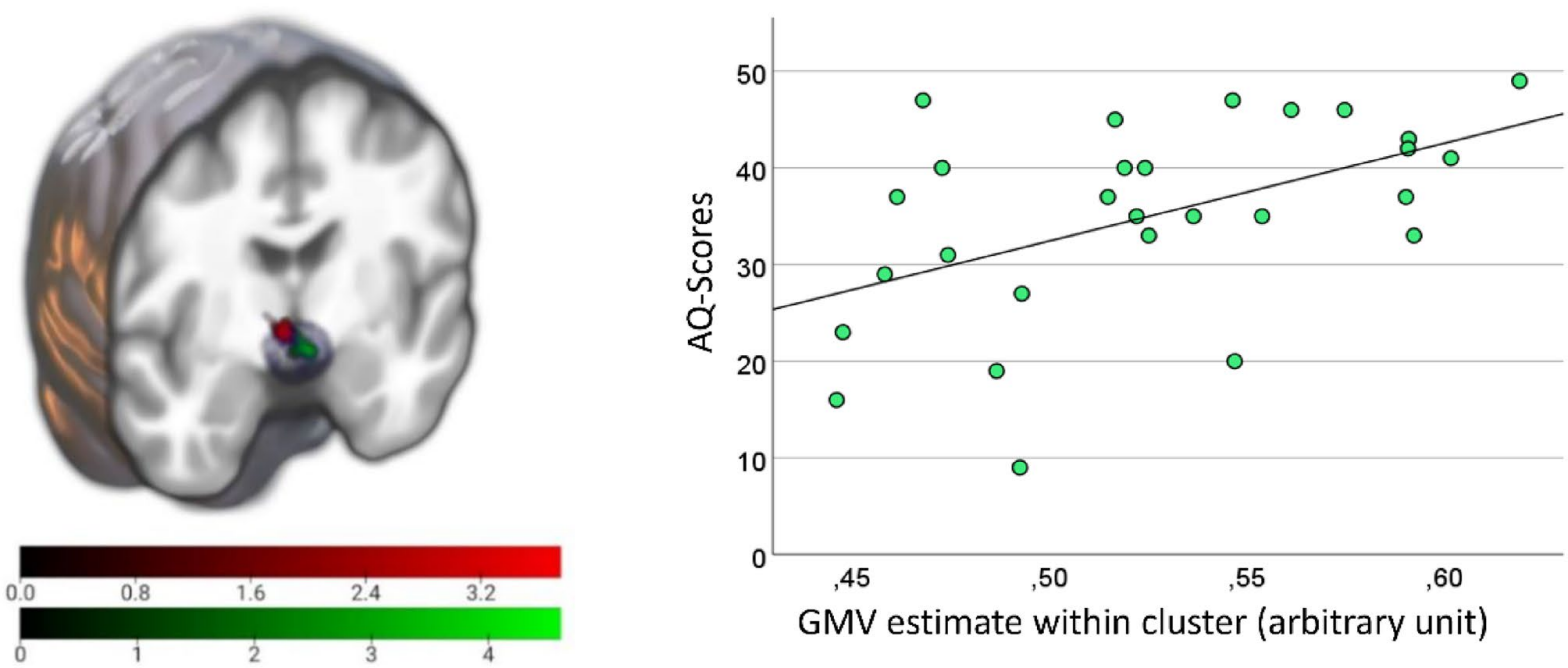
**Left**: Illustration of clusters from both association analyses and HTH outlines (grey). Green: association between GMV and AQ scores in the ASD group. Red: Cluster from previous analysis using OXT levels. **Right**: Scatterplot of the extracted GMV estimate within the cluster illustrates the positive association with AQ scores in the ASD group. *ASD* Autism spectrum disorder; *AQ* Autism spectrum quotient; *GMV* Gray matter volume.

There was no significant correlation between GMV and AQ scores within the HTH across the groups. Group-specific correlation analysis revealed a significant positively correlated cluster in the ASD group at peak MNI coordinates [ − 2,2,9] (FWE corr. *p* = 0.014, T = 4.74, Z = 3.92, k = 46) (Fig. 2), while there was no significant correlation in the CG. Likewise, repetition of the model using mean hypothalamic GMV showed a significant association in the ASD group (F(1, 23) = 12.78, *p* = 0.002, η^2^ = 0.357) while there was no significant correlation in the CG group (F(1, 22) = 0.215, *p* = 0.648). To explore if the cluster in the ASD group matched the cluster from the previous analysis including the OXT levels, the two clusters were plotted together on the structural mean image (Fig. 2, left). Visual comparison showed only a marginal overlap between the two clusters. Explorative correlation of the GMV cluster estimates with plasma OXT levels was not significant. Exploratory whole brain analysis revealed no significant findings in the hypothalamic region, but symmetrical clusters in both cerebellar hemispheres (Supplementary Fig. 2). No other region reached significance here.

## Discussion

Guided by previous research that has indicated potential links between OXT and ASD as well as between the HTH and ASD, the current study employed a hypothesis-driven approach to examine structural characteristics of the HTH and explore its possible link to peripheral OXT levels and autistic traits in autistic and non-autistic adults. Three main study aims were hereby addressed: First, we compared hypothalamic GMV between groups, but found no group-related differences. Second, we examined possible group differences in associations of hypothalamic GMV and peripheral OXT levels. Here, we found a positive association in the ASD group opposed to a negative association in the comparison group. Third, we examined a possible association of hypothalamic GMV with autistic traits. When we examined autistic and non-autistic subjects together, we did not observe any association, but upon conducting a separate analysis of the two groups, we found a positive association in the ASD group.

### *Differences in hypothalamic GMV*

Beyond the well-established central role of the HTH in regulating socio-emotional processes through OXT synthesis and release, evidence from both animal and human studies suggests a link between structural alterations in the HTH and ‘autistic’ behaviour. For instance, a study utilizing MRI-based neuroanatomical phenotyping to investigate twenty-six distinct mouse models for ASD consistently identified the HTH as one of the brain regions displaying abnormalities^55^. In line with this, Cntnap2 mutant mice, another mouse model for ASD, have been reported to display a reduction in the quantity of OXT immunoreactive neurons within the PVN of the HTH. Interestingly, the acute administration of OXT was found to ameliorate the social deficits observed in this mouse model^56^. Animal lesion studies in rats, cats and marmosets targeting the HTH have reported symptoms including antisocial and aggressive behaviors, as well as the suppression of sexual behavior^57–59^. Similarly, lesions of the HTH in humans, as seen in craniopharyngioma patients, have been linked to socio-behavioral and emotional impairments^27,28^. Despite this evidence indicating a close link between structural abnormalities in the HTH and some of the core symptoms of ASD, only a limited number of studies have explored its structure in autistic individuals. However, three studies have consistently reported decreased hypothalamic GMV observed in autistic children^34^, adolescents^32^ and young male adults^33^. Unlike these earlier studies we did not detect any group differences in our sample of adults. While our study is the first, to the best of our knowledge, to explicitly report no volumetric differences in the HTH between individuals with and without autism, it is important to note that whole-brain VBM is a method that does not rely on a priori spatial hypotheses. As such, VBM studies in participants with ASD that have found no abnormalities in the HTH could be interpreted as non-significant results with regard to this structure. However, as previously pointed out by Schindler et al. (2012), the sensitivity of VBM to changes in structures as small as the HTH depends largely on the hypothesis-dependent parameter settings^60^. Hence, a lack of discussion of the HTH in previous studies does not automatically imply a lack of effect in this region. While the large age range (18–60 years) of adults tested in our study may provide a good overview of robust structural effects, it risks overlooking age-specific effects. Taking into account the earlier research mentioned, which indicates that younger individuals with ASD may exhibit smaller hypothalamic volumes compared to control subjects, the lack of differences observed in our study involving adults may indicate a potential age-related normalization in hypothalamic GMV. Conducting an age-related subgroup analysis in our study was unfeasible due to the resulting reduction in sample size. Further, preferably longitudinal, studies are needed to test the hypothesis of age-related differences in hypothalamic GMV. However, it is worth noting that the notion of intricate growth patterns in regional brain volumes in ASD, which may be particularly prominent during specific phases of neurodevelopment, is not a novel concept^61–63^. For instance, prior research has indicated a growth pattern characterized by volume reduction followed by a convergence toward normal volume in the striatum^64^. Conversely, a growth pattern marked by age-related volume decrease has been proposed for structures such as the amygdala^65–67^. The mechanisms underlying these structural changes and whether they reflect a primary inherent growth pattern in ASD or possibly represent secondary compensatory adaptations remain unclear^68^. Based on the findings presented here we will discuss OXT as a potential factor underlying the apparent volumetric normalization of the HTH in autistic adults in the following section.

### A link between the HTH and OXT in autism

The involvement of OXT in regulating socio-emotional behavior has been extensively demonstrated across a wide range of studies in humans and animals^69^.The notion that differences in the OXT system might contribute to the core symptoms of ASD was initially introduced over two decades ago^70^. Human research has since brought to light various potential variations within the OXT system associated with ASD, encompassing areas such as the processing of OXT peptides^71^, genetic variations within the oxytocin receptor gene (OXTR)^72^ and the structural gene responsible for OXT (neurophysin-I)^73^, and epigenetic modifications^74,75^. Another frequently examined indicator of an altered OXT system in ASD is the concentration of peripheral OXT. Following the initial observation of reduced basal OXT levels in children with ASD^76^, numerous studies mainly in pediatric populations have yielded diverse and partially incongruent findings^77^. The data are particularly inconclusive for adults. While there are reports of lower^78^ and higher levels^79^ in autistic adults, our group recently added to the existing literature by reporting no differences in basal OXT levels^38^, which was also true for the subsample included here. In accordance with this finding and highlighting the importance of age in this context, two recent meta-analyses have concordantly reported basal concentrations of OXT to be lower in autistic children, while showing no significant differences in autistic adults when compared to controls^77,80^. This is suggestive for relevant developmental changes in the OXT system in ASD and possibly for a normalization of OXT levels in adulthood. The importance of developmental effects in this regard has also recently been shown with regard to OXTR expression patterns^81^. In the present study, we have found indications that raise the possibility of age-related normalization in OXT levels potentially translating into normalization in hypothalamic GMV. This hypothesis finds support in the observation that, despite the absence of volumetric differences in the HTH between the groups, we found a positive association between peripheral OXT levels and hypothalamic GMV among autistic adults when compared to non-autistic adults. Additionally, the exploratory whole-brain analysis indicated that the cluster was statistically significant not only within the HTH but also extended to the thalamus, with no other regions showing statistical significance in this context. Although the latter observation warrants further validation in larger samples, it aligns with reports suggesting a potential involvement of the thalamus in OXT release in the HTH^82^. Partially aligning with our findings, prior research has indicated a positive relationship between VP and hypothalamic GMV. However, it's worth noting that this study did not include a comparison of this association with a control group^34^. The regulatory influence of OXT on neuronal plasticity and its involvement in both inhibitory and proliferative cellular processes in specific regions, including the HTH, has been firmly demonstrated in both animal and human studies^83–88^. Hence, it seems plausible that the structural properties of HTH are related to OXT levels. Taking into account earlier research on OXT levels and structural analyses of the HTH in ASD, our results suggest the possibility of a (compensatory) increase in OXT production after childhood, potentially aligning with an increase in GMV within the HTH. As a result, this mechanism could potentially contribute to the normalization of OXT levels and hypothalamic volume in adulthood. While a straightforward explanation might involve an increase in the volume or number of OXT-producing cells, the precise tissue characteristics responsible for the observed GM signal within the HTH in VBM studies, including our own, remain uncertain. Beyond cell volume, factors such as nuclear volume, local cell count, and the spatial arrangement of neurons, glia, blood vessels, and neuropil could potentially contribute to these alterations^89^. Remarkably, variations in all these morphological and cytoarchitectonic features have been documented in various brain regions in autistic individuals^90^. To this point, it remains unclear whether the here observed associations in the HTH are directly attributable to OXT or mirror other factors related to peripheral OXT concentrations. For example, it remains controversial whether and to what extent peripheral OXT measurements can effectively mirror the central regulation or dysregulation of the OXT system. In particular, a coordination of peripheral and central OXT levels at baseline conditions has been called into question^91^. The inconclusive picture regarding the informativeness of peripheral OXT levels has prompted researchers to investigate genetic variation in the human OXTR gene. In this context several single nucleotide polymorphisms (SNPs) such as rs53576 and rs2254298 have been linked to ASD^72^. Interestingly, Tost et al. reported an association of these variants with structural differences in the HTH in healthy carriers^35,36^. Furthermore, rs2254298, along with other OXTR variants with increased likelihood for ASD, have been reported to be closely linked to peripheral OXT levels^92^. This raises the question of whether changes in the HTH and OXT levels are modulated by common factors such as variations in OXTR. It also remains to be clarified to what extent other genetic variations of the OXT system such as the structural gene for OXT (oxytocin-neurophysin I) and CD38 (associated with OXT release) play a role^93^.

### A link between the HTH and autistic traits

Behaviour, including social behaviour has been shown to affect structural properties of the brain^94,95^. The AQ measures autistic traits in both autistic and non-autistic individuals and has been shown to correlate with overall GM variations in autistic individuals^96^, as well as with multiple metrics of regional GM including volume, cortical thickness, surface area, gyrification and cortical thickness in autistic as well as non-autistic people^97–99^. However, none of these previous reports includes the HTH. As mentioned above, for reasons specific to the technique of investigation, the absence of reports in these studies on a structure as small as the HTH does not automatically imply an absence of effects in this region. Expanding upon these prior reports, our initial analysis involved examining correlations of hypothalamic GMV and autistic traits across autistic and non-autistic participants. This analysis did not yield statistically significant outcomes. Subsequent group-specific analysis, however, showed a positive correlation in the ASD group, whereas no such correlation was evident in the CG. This finding suggests a potential link between the HTH and autistic traits in ASD, while also hinting at individual differences in this regard. It is important to note that the interpretation of this finding is somewhat constrained by the fact that the subsequent exploratory whole-brain analysis did not yield significant results in the hypothalamic region. This raises questions about the regional specificity of this finding, highlighting the necessity for further in-depth investigations. As is generally the case in VBM association studies, a causal interpretation of the nature of this association (increase in GMV alongside heightened autistic traits) is only possible to a limited extent. Ecker et al. (2012) have previously emphasized the need for extensive longitudinal studies to differentiate neuroanatomical changes primarily associated with the disorder from those that might occur as secondary, possibly compensatory mechanisms^68^. Given the composition of our sample of adults with HFA, it is conceivable that the observations made here reflect the *result* of an atypical brain development rather than representing primary neuropathological characteristics of ASD. Assuming a secondary causation in these findings, the observed correlation could imply a compensatory growth of the HTH in response to increased autistic traits. Consistent with this hypothesis, one might speculate that there exists a intricate interplay between OXT release and behavior, with potential repercussions on observable brain structural changes in the HTH^100^. Building upon this idea, we tested whether the hypothalamic area associated with autistic traits (as indicated in model 3) might also show a link to OXT (as per model 2). However, subsequent exploratory analyses did not uncover any computational or visual correspondence between the two analyses. Furthermore, there was no significant correlation between peripheral OXT levels and AQ scores. The inconsistent findings in previous research in this regard underscore the limitations of a simplistic OXT deficit or excess model for ASD. For example some studies have shown no correlation between OXT levels and autistic traits^79,101–103^ while others have even reported on a negative correlation between higher OXT levels and social skills in the autistic population^76,104–106^, indicating greater social impairment with elevated OXT levels in autistic individuals. This counterintuitive finding has been hypothesized to reflect broader abnormalities at multiple levels of the endocrine OXT system, including the OXT gene, disruptions in the processing of the OXT molecule, and OXTR abnormalities that result in compensatory but insufficient increases in OXT levels^104^. Based on this hypothesis, it seems plausible that the observed positive correlations within the ASD group between hypothalamic GMV and both OXT levels and AQ scores could be construed as a compensatory phenomenon. Once more, additional research is required to elucidate the intricate interrelationships, particularly concerning the variations in OXTR and their association with OXT levels in ASD.

### Implications for clinical practice and research

Presently, no approved pharmaceutical treatments exist for the core symptoms of ASD, and reported findings regarding the efficacy of OXT are inconsistent in both child and adult participants^107^. The shortcomings of clinical trials in OXT pharmacotherapy can be attributed to various factors, including a limited understanding of the biological basis of ASD, the absence of clinically meaningful markers to identify homogeneous patient subgroups, and the consequent absence of targeted therapeutic options^16^. The results of this study point to a potentially important role of the HTH as a neurobiological correlate of ASD. However, further investigation is needed to assess the clinical relevance of these findings, particularly with regard to how structural and functional changes in the HTH manifest in ASD over a lifespan and whether alterations in the HTH can help characterize ASD subtypes. Further exploration of the intricate connections between genetic variations in the OXT system, morphofunctional alterations in the HTH and associated behavior seem essential in this context. Furthermore, obtaining a more comprehensive understanding of the structure and function of the HTH in ASD may have broader implications for other psychiatric conditions characterized by socioemotional impairment. For example, a study conducted by Mielke et al.^112^, using a methodology similar to the one employed here, investigated women with a history of early childhood maltreatment. Their hypothesis-driven approach was centered on the notion that deficits in reward processing in adults who experienced childhood maltreatment might be correlated with OXT levels and structural alterations in the HTH. Intriguingly, compared to our findings, their findings revealed a contrasting relationship between peripheral OXT levels and HTH volume in patients versus controls, suggesting the possibility of a distinct association between OXT and the HTH compared to ASD. Although it is premature to draw conclusions from that, conducting comparative analyses of the relationship between OXT and the HTH across various psychiatric conditions presents a promising avenue for future research. In addition to craniopharyngioma patients mentioned above^108^, hypothalamic abnormalities have also been reported in schizophrenia^109^ and mood-disorders^60^. Given the heightened prevalence of depression in individuals with ASD, a comparative analysis between depressive and autistic people would be particularly relevant to assess the specificity of structural effects in the HTH.

### Limitations

The results presented here should be interpreted cautiously and in the context of some methodological considerations and limitations. Given the well-established role of the HTH for the OXT system and the potentially important role of OXT in ASD, a surprisingly small number of studies have focused on this brain structure. This is partly due to the methodological difficulties in proper identification of the HTH in MRI images. Here, we took a relatively straightforward approach by using a HTH mask created on the basis of 168 typical adults^53^. Since this mask was not created specifically for our sample, it can only be considered a rough regional reference. Manual delineation remains the gold standard, but comes at the cost of a high degree of expertise and time investment^52,110^. Other studies have used a HTH mask based on the WFU pick atlas^111,112^, which we decided against since it covered the HTH much less accurately based on visual comparison. Currently, none of the atlases implemented in Cat12, such as AAL3 or Neuromorphometrics, include the HTH, which prevented us from using automated ROI analysis in the native subject space^113^. The implementation and improvement of such atlases as well as the use of deep-learning approaches^114^ are promising developments in terms of accurate and user-friendly volumetry in this brain region for future studies. Further limitations concern sample characteristics: In our study we included autistic adults with HFA. This naturally limits the generalizability of findings to the entire autism spectrum. However, this limitation may also prove advantageous, as there are reports suggesting a strong link between hypothalamic abnormalities and intellectual impairment^115^. This association could pose challenges in attributing findings in the HTH solely to the autistic phenotype in autistic individuals with intellectual impairment. Although the groups were not significantly different, they were not optimally balanced in terms of age and sex distribution. Particularly in light of reported sex-dependent differences of the HTH in neurotypicals^116^ and in associations with OXTR^35,117^, this might warrant sex-specific analyses in a larger sample. Furthermore, while we corrected for sex and age in all analyses, we did not account for possible differences in OXT levels with respect to post-partum effects in women aside from the exclusion of pregnancy and breastfeeding. While large increases in OXT levels around parturition are known, the temporal extent to which these post-partum changes are detectable is poorly studied^118^. In this sample, 45% of autistic participants received psychiatric medication on a regular basis. This adequately represents the high degree of comorbidities in autistic adults in terms of a naturalistic study design^4^. Due to the various pharmaceutical substances (antipsychotics, antidepressants, stimulants) and different dosages, it did not seem feasible to include medication as confounding variable. While the impact of psychopharmaceuticals on brain structure has been shown in a range of studies, studies to date have not reported on specific structural alterations in the HTH due to medication in this regard^119,120^. Given these limitations, the results reported here should be interpreted with caution and warrant further validation in a larger and ideally unmedicated sample.

## Conclusion

Bearing in mind the limitations, this study provides evidence that hypothalamic GMV does not differ between autistic and non-autistic adults. Although this study does not provide insight into a causal relationship, findings further suggest a potentially important role of the HTH in relation to OXT and autistic traits in ASD. Moreover, our results underscore the relevance of individual variations in this context. Taking previous research into account, our findings raise new questions about possible developmental changes in the structure of the HTH and its link to OXT in ASD, encouraging further exploration. Specifically, a better understanding of the interplay between genetic variations in the OXT system, OXT levels, and brain structure could significantly enhance our understanding of OXT's role in ASD, both as pathophysiological factor and potential therapeutic agent.

### Supplementary Information

Supplementary Information.

## Data Availability

The data that support the findings of this study are available from the corresponding author, RH, upon reasonable request.
