# Supplementary material for: Peripheral oxytocin levels are linked to hypothalamic gray matter volume in autistic adults: a cross-sectional secondary data analysis
Source: Sci Rep. 2024 Jan 16;14:1380. doi: 10.1038/s41598-023-50770-5 (PMC10791615; doi:10.1038/s41598-023-50770-5)
Supplement: Supplementary file 1 — Supplementary Information. [file 41598_2023_50770_MOESM1_ESM.docx]

# Supplementary Information

## Quality check of MRI images

Prior to pre-processing, images were visually examined for scanner artefacts and correct alignment using the SPM display function. Hereupon one individual was excluded due to visible stripe artefacts in the image, probably due to head movement during recording. Another individual was excluded due to visible anomalies in the temporal lobe. In accordance with clinic guidelines, the individual in question was recommended to consult a neurologist. In a further step, all scans underwent an automatic quality assessment in Cat12, based on quality parameters such as noise, image inhomogeneities and resolution, which resulted in an Image Quality Rating (IQR) from 0 (unacceptable quality) to 100 (excellent quality). IQRs for each scan were extracted and implemented in SPSS for group comparison (ASD vs CG) using an independent two sample t-test to exclude significant group differences regarding image quality. After segmentation, but before smoothing, followed a series of quality checks to identify any inconsistencies that might have occurred during pre-processing. First, all GM images were visually inspected individually for gross pre-processing errors. In a second step, one horizontal slice from the normalized, bias-corrected and modulated scans of each subject was visualized together on one page using Cat’s function ‘display slices’. This was done to facilitate the visual identification of potential gross segmentation errors. As a final step, Cat’s function ‘quality check’ was carried out, which offers visualisation of the overall correlation of the images in the form of a violin plot and a correlation matrix, thus, facilitating the identification of potential outliers. Images with a low overall correlation were again inspected carefully. As an additional feature, the Mahalanobis distance between ‘mean correlation’ (reflecting the quality after pre-processing) and ‘image quality’ (reflecting the quality before pre-processing) was plotted as a measure of overall combined image quality before and after pre-processing. As a result of the quality check protocol, one outlier regarding overall correlation and image quality was identified, clearly distinguishable in the Mahalanobis distance plot. There are no clear criteria as to when a scan based on these quality measures should be excluded if no obvious artefacts are found. Although no obvious artefacts or anomalies could be identified visually, it was decided to exclude this scan from further analysis to avoid biased results. In summary, three scans were excluded from further analysis in accordance with the quality check protocol. The remaining 56 images had a mean quality rating of 85.3 (with a range of 82.7 to 86.37), reflecting good overall image quality. The mean quality rating in the ASD group was 85.4 and mean quality rating in the CG was 85.2. A two tailed two sample t-test confirmed that the quality scores did not differ significantly between groups (*p= 0.62*).

## Quantification of plasma OXT

Procedures have been previously reported in (1). Plasma was taken into blood collection tubes (Sarstedt, S-Monovette K3E 2.7 ml or 7.5 ml, Cat.nr.: 01.1605.001). Immediately after blood collection the tubes were transported in a cooling box. Then plasma was centrifuged at 4 **°**C for 15 min with 2500 × g. The supernatant was filled into a 4 ml tube (Sarstedt, 92 × 15.3 mm, PP, Cat.nr.: 62.611) and centrifuged again. After centrifugation the samples were aliquoted into 2D-barcode tubes (Brooks, fluidX, Cat.nr.: 68-0703-12) and stored at -80 **°**C. Plasma OXT concentrations were quantified by an external laboratory (RIAgnosis, Sinzing, Germany) using radioimmunoassay (RIA) as previously described (2). The analysis was performed on encoded samples without providing any additional information (including times of sample collection, matching pairs of saliva and plasma etc.). According to the provider plasma samples (0.5 ​ml) were kept at −20 ​°C until extraction using LiChroprep® Si60 (Merck) heat-activated at 690 ​°C for 3 ​h. 20 ​mg of LiChroprep® Si60 in 1 ​ml distilled water were added to the sample, mixed for 30 ​min, washed twice with distilled water and 0.01 ​mol/l HCl eluded with 60% acetone and evaporated. Following evaporation (Concentrator, Eppendorf, Germany) 50 ​μl of assay buffer was added followed by 50 ​μl antibody raised in rabbits against OXT. Finally, after 60-min pre-incubation, 10 ​μl of 125I-labeled OXT was added. The detection limit of the RIA was in the 0.1–0.5 pg/sample range depending on the age of the tracer. Intra- and inter-assay variabilities were < 10% and cross-reactivities with related peptides < 0.7%. All samples were assayed in the same batch. Serial dilutions of samples containing high levels of endogenous OXT run strictly parallel to the standard curve indicating immuno-identity.

## Exploratory whole brain analyses


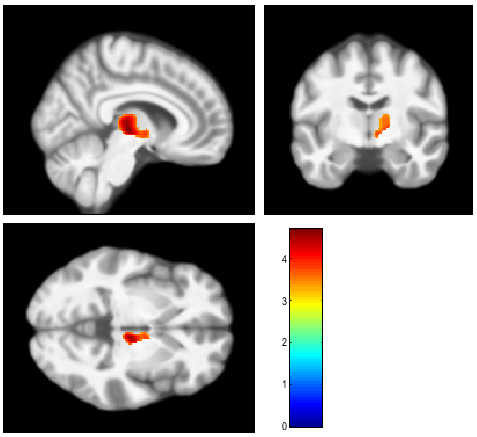

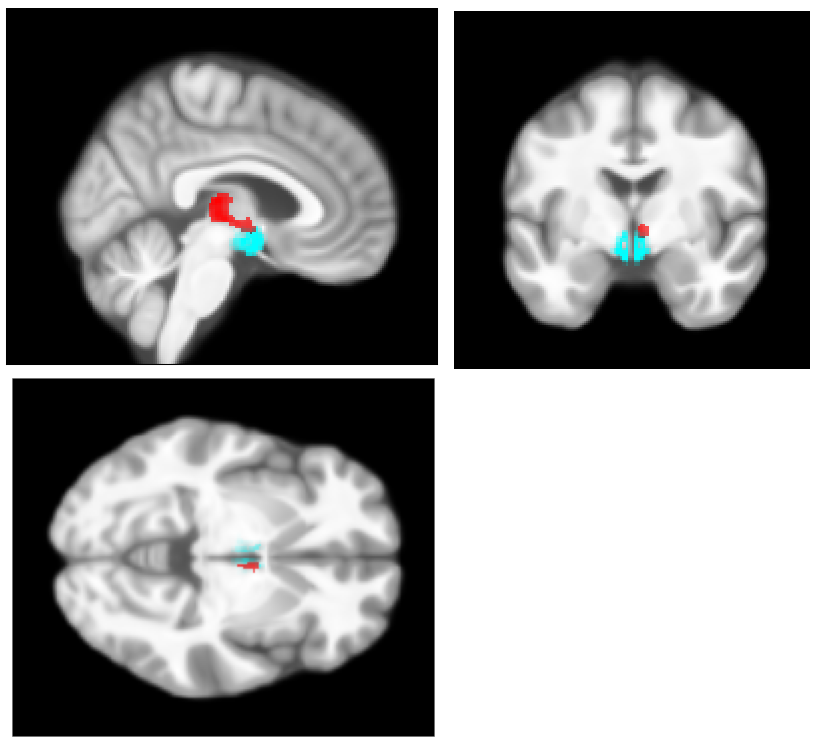


Supplementary Figure 1. (A) Exploratory whole brain analysis for the contrast (GMV[ASD] × OXT > GMV[CG] × OXT) including sex, age and TIV as control variables revealed that the HTH cluster was part of a larger cluster (FWE corr. p=0.005 , T= 4.69, Z=4.23, k=1373) extending from mainly the right thalamus to the HTH. (B) Synopsis of the larger cluster (red) and the HTH mask (cyan) at the section, where both overlap.

A(FWE corr. p=0.005 , T= 4.69, Z=4.23, k=1373)

B

*
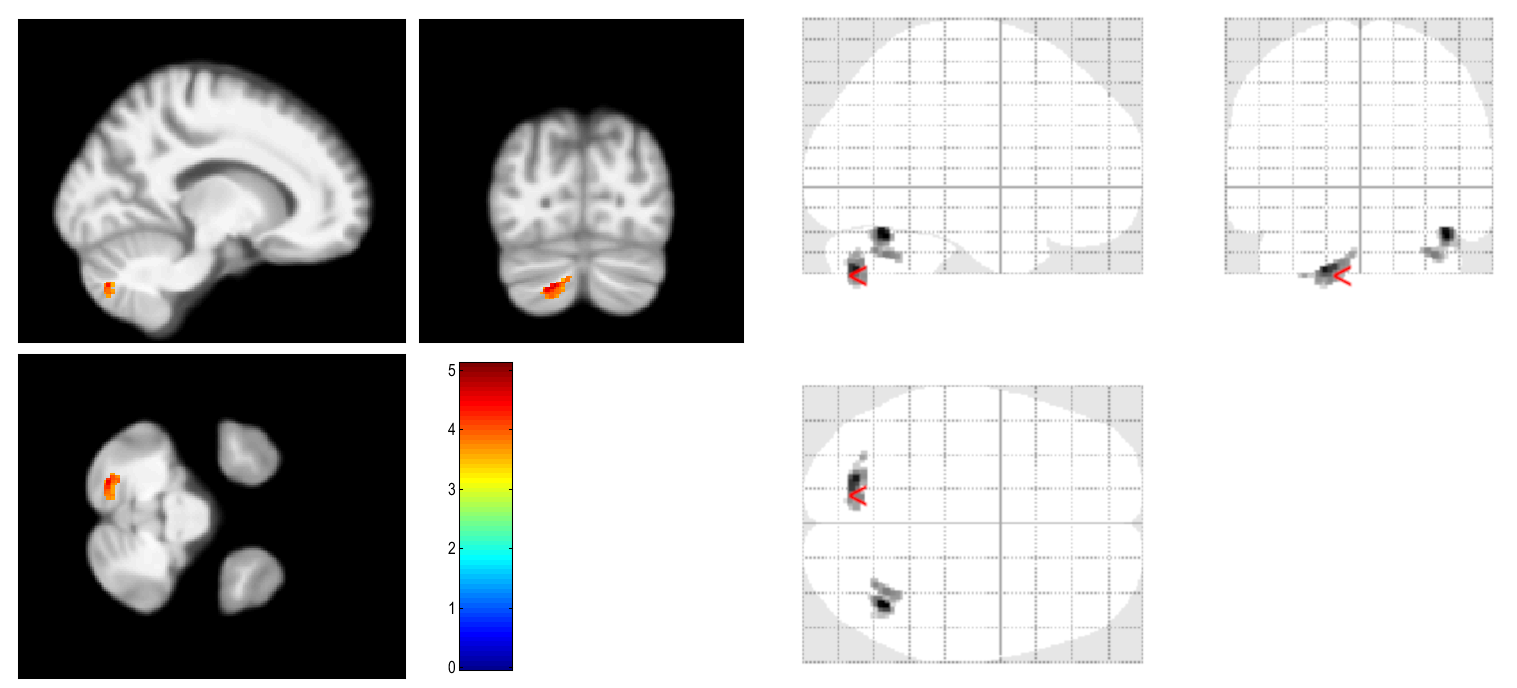
*

Supplementary Figure 2. Exploratory whole brain analysis for associations of GMV with AQ scores in the ASD group including sex, age and TIV as control variables. Left: Clusters overlayed on mean structural image. Right: glass brain view of the same clusters. A positive contrast revealed one cluster in each cerebellar hemisphere at peak MNI coordinates [42,-60,-26] (FWE corr. p=0.031, T= 5.08, Z=4.12, k=184) and [-20,-75,-42] (FWE corr. p=0.009, T= 4.88, Z= 4.00, k=232). There was no significant cluster in the hypothalamic region. No other region reached significance here. There were no significant clusters for a negative contrast.
